# Strangulated Umbilical Hernia—Herniotomy—Cure

**Published:** 1882-04

**Authors:** Herman Mynter


### Clinical Reports.

### STRANGULATED UMBILICAL HERNIA—HERNIOTOMY—CURE.

Reported by Herman Mynter, M. D.

Mrs. S., 54 years of age, has for 19 years occasionally had a protrusion at the umbilicus, followed with symptoms of obstruction of the bowels, but has always been able to bring it back again, although she thinks, that it never has been perfectly reduced. February 2, 1882, at 5 P. M., the hernia came out, and she was unable to reduce it. During the evening a physician was called, who tried taxis under narcosis with partial success, some gurgling sound being heard and the protrusion being diminished in size. Calomel and morphia and large enemata were ordered, without being followed by movements of the bowels. During the night frequent vomiting occurred, continuing during the next two days. February 5th Dr. Rochester was called in, and finding the vomiting of stercoraceous nature, advised operation and referred the patient to me.

By the examination, at 9 P. M. February 5th, the patient was feeling quite comfortable; temp. normal; p. 84; tongue clean; skin natural. A protrusion was felt beneath the navel as large as two fists, of rather soft consistency, with dull percussion over most of the surface. The navel itself was protruded farther than the rest. The skin over the tumor was of natural color; very little pain of tumor or abdomen by pressure. Bowels had not moved by injection. She had not vomited much for several hours, and it was therefore decided to defer the operation till next day. All nourishment was stopped during the night, only ice pills being allowed, and the tumor was covered with an ice-bladder. During the night and following forenoon, February 6th, copious stercoraceous vomiting occurred, and the operation was therefore performed at 3 P. M., four days after the accident, Drs. Tobie, Diehl, Mann and Bartow being present; pulse 96, rather full, temperature 99, no particular pain in tumor or abdomen.

Under ether-narcosis taxis was first tried, and this failing, herniotomy was performed. An incision, two inches long, was made in linea alba over upper half of tumor, where the ring was supposed to lay. Immediately under the integuments an old protrusion of omentum, slightly congested, as large as two fists, was found, adherent with old, but easily ruptured, adhesions to the sac. The incision was therefore extended downwards two inches to give place. After the adhesions had been severed, a single coil of the small intestines was found, perfectly covered by the omentum. It was considerably congested, but otherwise of sound appearance and feeling, and was returned with ease after the ring, which scarcely admitted one finger, had been incised slightly downwards. The coil was adherent to the inner surface of the ring. The torn and bleeding omentum was double ligated with catgut and cut off, as it, on account of adhesions all around the ring, could not be returned. The sac was thoroughly cleaned with carbolized water, and two silver wire-sutures introduced through the whole abdominal wall and peritoneum to close the ring and prevent any entrance of matter into the abdominal cavity. The upper angle of the wound, where the stump of the omentum was situated, and the lower angle were left open for drainage. Lister's antiseptic gace was applied as dressing, and morphia in ¼ grain doses ordered as often as necessary.

February 7th. Temp. $\frac{100}{99}$, pulse $\frac{108}{96}$; no vomiting, pain or symptoms of peritonitis.

February 8th. Very restless during the night in spite of morphia (gr. 1 in 4 doses). Several times greenish vomiting. Pulse 100, temp. 100. Some offensive discharge at lower angle of wound.

February 9th. No vomiting; no tenderness of abdomen; bowels moved; feels quite well.

February 12th. The silver sutures were removed, having cut through the skin.

February 24th. Wound almost healed. A little, deep-seated, hard lump felt to the right of the wound, painful by pressure.

February 25th. Discharge through the wound of badly-smelling pus by pressure on the lump, which feels softer. Incision with evacuation of 2 ℥ pus.

March 5th. The wound healed, the patient is up and feels well. The cicatrix is hard and retracted, no impulse by coughing. The ring cannot be felt, and only a little hardness is felt where the stump of the omentum is situated.

A strangulated umbilical hernia has always been considered extremely dangerous, especially on account of the usually large size of the protrusion and the quickness with which mortification of the bowels occurs, owing to the hard, unyielding ring in which the strangulation generally takes place. Uhde (Bilroth & Pitha's Surgery) gives the result of 122 herniotomies, of which 55 died, 67 recovered. In the case mentioned here the large protrusion of omentum undoubtedly acted as a cushion and prevented the quick mortification which usually occurs. A point of interest is the wiring together of the ring, to prevent matter from the large sack and the stump of the omentum from entering the abdominal cavity, by producing an adhesive inflammation. So far the result is perfect, as no impulse is felt by coughing. In regard to the omentum, it must be considered safer to cut it off and have the stump outside the peritoneal cavity, than to loosen the adhesions and return the bleeding, torn and congested mass.

